# An Interview

**Published:** 1898-03

**Authors:** 


### An Interview.

BY NEMO.

It was a dull day, and rainy. The clouds depressed one's spirits. Practice, too, was not at all what might be desired the non-payers calling quite faithfully to promise, while the better class held aloof. I was somewhat dejected, indeed gloomy, for many things had gone wrong of late, and I, one of the poor, seemed to be illustrating the current saying about the rich becoming richer and the poor poorer. My reverie was interrupted by a step, quick and elastic, and I was in the presence of a former student, with a very cordial "howdy" and a smile as bright as could be.

"Glad to see you," I exclaimed, and I really was, for I am always brightened by a visit from one of the "boys," and this visit came quite welcome, inasmuch as I was needing a cheerful visitor. (Mem.: The landlord's clerk had been up shortly before to ask for "that little balance due from last month.")

"Glad to see you, Professor; mighty glad; it looks quite like old times to see you again. How have you been? Well, I trust. And busy, of course."

I gave an evasive answer about the "busy," for things, as I intimated, were not as bright as might be, but I braced up and asked my visitor about himself.

"Doing splendidly, Professor; I have done as well as any young man, I dare say, who started out in the world with as little as myself. I have no cause of complaint. I am established in the town of Take-em-in, and I have as pretty a practice, I suppose, as any one in the State. I have a fine office, with two assistants, always busy, and we wait on as many people, I suppose, in a day, as three men anywhere. My employees are first-class operators, both of them college graduates, and they only cost me $12 00 a week each. They board themselves. I could get men who are first-class operators, for a third less, but they are not sober men."

"What are your average fees?" I asked.

"Well, for amalgam fillings we get fifty and seventy-five cents, platinum fillings cost more—say from one dollar to two. Plates? Oh, well, three dollars is the lowest for a full upper set, and we get out a good many. I suppose a good man can make four or five sets a day.

"Of course I have to do a good deal of advertising, but as a rule I find it don't pay to advertise in the newspapers. The best ad is on the theater posters. I have a big lithograph tooth, as tall as a man, and when this is illuminated at night it draws quite a deal of attention. A wagon with a big sign on it might not be bad, but we havn't tried that yet. A big thing is an electric light for illuminating the mouth. It always attracts attention. We call it an X-Ray light, and it looks very scientific.

"I have another office down in the lower end of the county, and keep a man going there. It isn't as good paying a place as at home, but it keeps business going. You see, we must hustle nowadays, or we'll get left.

"I had some trouble with this county office. Some officious dentist or other wanted to haul me up before the court for violating the law regulating the practice of dentistry. I was employing a man who wasn't a graduate. But the court soon disposed of that question by a decision that what a man did by his agent he did himself, and my employee was my agent. It is quite astonishing how little law those dental boards know."

"So you like it out there, with that sort of practice?" I remarked.

"Oh, yes," was the enthusiastic reply. "I think it just grand.

I tell you Professor, I wouldn't give my profession for any other I ever heard of. I just love it."

"But," I mildly asked, "do you think that sort of practice is exactly professional? Isn't it a little--er, irregular?"

"Oh, well now, Professor, you know business is business, and when you are in Rome you must do like the Romans. If you want to make money you must hustle for it nowadays, those old fashioned ways are clean out of date now. The truth is Professor, you know that in the mechanic arts the only way to make anything is to have employes, and get the profits of their labor. Why, dear me, if I had to work at dentistry the old fashioned way, I wouldn't make my salt. As it is I get the profits on the labor of my employes, and the harder I push them the more there is for me, and I take it easy for myself; at all events, I am looking forward to the time when with two or three establishments I will be able to lay back and simply direct matters."

"But what of the quality of the work?" I suggestively asked.

"Well, Professor, I think for the pay we get, we render a fairly good quality of work. You see, it's just like any other business, conducted on business principles. If a man wants two dollars worth of work he gets it, and if he is fool enough to think that he can get the best in the office for fifty cents, why he's mistaken. It's business, you see, like any other business. We do the best we can for the money, and people should understand that cheap things are—well, cheap. I should be a great fool to put, or allow one of my operators to put, two dollars, worth of work on a fifty-cent job. It isn't business.

"Yes, the profession is a great thing, the greatest thing in the world. I feel proud of it and grow to love it more and more as I see its beauties."

